# Polymeric Membrane Electrodes for a Fast End Cost-Effective Potentiometric Determination of Octenidine Dihydrochloride in Pharmaceutical Samples

**DOI:** 10.3390/ma18174100

**Published:** 2025-09-01

**Authors:** Joanna Lenik

**Affiliations:** Department of Analytical Chemistry, Institute of Chemical Sciences, Faculty of Chemistry, Maria Curie-Sklodowska University, M. Curie-Sklodowska Sq. 3, 20-031 Lublin, Poland; joanna.lenik@mail.umcs.pl

**Keywords:** ion-selective electrodes, solid contact, octenidine determination

## Abstract

Determining the active substance content in the tested product is an essential part of research for overall assessment of the quality of a medicinal substance. This role can be successfully performed by membrane electrodes that are selective for a specific drug. The novelty of the presented research is the development of the first ion-selective electrode with a polymer membrane phase with the octenidine (OCT) function. Classical ion-selective electrodes (ISE), polymer electrodes with an internal Ag/AgCl electrode, and electrode bodies with glassy carbon were used for the research. The membranes were prepared based on cation exchangers from the borate group and neutral cyclodextrin. All sensors have good parameters, e.g., the polymer electrode with KtpClPB is characterised by a wide linear range of −logc 6−3, a low limit of detection 5 × 10^−7^ M, and a near-Nernstian, reproducible slope of characteristics of 31.41 ± 1.14 mV/decade. It can be seen that a stable, reversible potential and a short response time were achieved for this sensor. The obtained favourable selectivity coefficients of the electrode determined in relation to excipients allowed direct determination of octenidine, e.g., in lozenges. The results obtained with the calibration curve method show a recovery of 97% and a precision of SD 2.3 mg/L, which indicates that the data are consistent with the pharmacopoeia requirements.

## 1. Introduction

The issue of drug quality control is a very important and still relevant topic, especially given the rapid development of the pharmaceutical industry and the emergence of a large number of new medicinal products. Every medicinal product on the market must be of sufficient quality to guarantee its safety and efficacy. The global organisation responsible for harmonised standards for the quality, efficacy and safety of medicines is the International Council on Harmonisation of Technical Requirements for Registration of Pharmaceuticals for Human Use (ICH). This organisation is also supported by the World Health Organisation (WHO). In the case of European Union countries, ICH guidelines are most often presented as EMA (European Medicines Agency) documents. The determination of the active substance content in the examined product is an essential part of overall quality assessment of a medicinal substance. For this purpose, chromatographic methods coupled with mass spectrometry (HPLC-MS, GC- or instrumental endpoint detection (potentiometric, amperometric, voltammetric)) are most often used. The selected analytical methods should guarantee an objective and reliable assessment of the results obtained in a relatively short time and at low analysis costs. Analytical methods should be precise, accurate, selective, sensitive and have a low detection limit. Any difficulties and errors that arise during quantitative research may be related, for example, to the nature of the sample matrix and the occurrence of various interferences, the type and stability of the analysed substance, and its concentration. Therefore, as the pharmaceutical industry develops and new drugs are created, analytical procedures require the introduction of new developments, continuous updating, or refinement of existing procedures [1,2].

One of the most popular groups of drugs, according to pharmacological classification, are medicinal products that act against pathogenic microorganisms. These include, among others, antiseptics, i.e., agents that disinfect living tissues of the human and animal body, both external (i.e., hair, skin, nails) and body cavities (e.g., oral cavity, nasal cavity, larynx, throat). These substances have specific properties and must act relatively mildly in order to maintain the physiological integrity of the disinfected tissues. Their activity against microorganisms, i.e., bacteria, viruses, fungi, and protozoa, is associated with their bactericidal (or bacteriostatic) effect causing a complete destruction of microorganisms, e.g., by damaging cytoplasmic membranes, denaturing microbial proteins, reacting with nucleic acids and microbial enzymes, and causing reactions disturbing the surface tension of microbial cell fluids or oxidation reactions. Another type of action involves inhibiting the growth of microorganisms and preventing their transmission [1]. A group of cationic surfactants (CS), which includes quaternary ammonium salts such as benzalkonium salts, cetylpyridinium salts as well as derivatives of tertiary amines such as octenidine, plays a special role in medicine and pharmacy. They have an important function, e.g., in biotechnological applications, in nanotechnology [3,4,5] as drug carriers, as antibacterial agents and bioimaging agents, and stabilisers of nanoparticles and nanocarriers.

In the studies presented in this paper, the active substance was octenidine dihydrochloride. It is a substance used in well-known pharmaceutical preparations, i.e., two-component preparations such as Octenisept, Ophenic, Linoseptic, and Maxiseptic in the form of liquids and sprays for use on the skin [6] and single-component preparations such as Octeangin, Laryngomedin, and Octenidin, most often in the form of lozenges [7].

**Figure 1:**
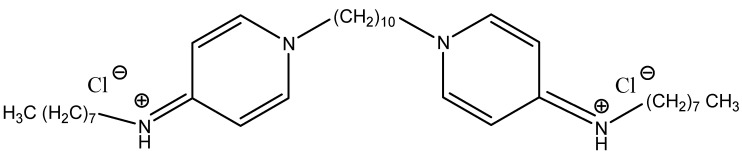
Octenidine dihydrochloride, 1,1′-(Decane-1,10-diyl)bis(*N*-octylpyridin-4(1*H*)-imine)—hydrogen chloride.

Octenidine was synthesised in the early 1980s and underwent clinical trials, as a result of which it successfully passed in vivo and in vitro tests [8]. It is a tertiary aliphatic amine derivative containing two cationic centres that enable it to bind to negatively charged components of microbial cell membranes, leading to their destabilisation, followed by disintegration and death. The molecular formula of octenidine dihydrochloride is shown in Figure 1.

It is a white-to-off-white powder, soluble in solvents, i.e., dimethyl sulfoxide and methanol, and its solubility in water is 14.2 g/L at 20 °C, although the dissolution rate can be increased by using an ultrasonic bath. In clinical studies, octenidine has been shown to be the most effective active agent of many antimicrobial substances tested, among others triclosan, polyvinylpyrrolidone-iodine, polyhexamethylene biguanide, and chlorhexidine digluconate (CHX), using a minimum effective concentration and a minimum bactericidal concentration [9,10]. Pharmaceuticals containing octenidine are mainly used for skin surface disinfection and wound decontamination as well as for acute pharyngitis. The spectrum of action of octenidine is very broad and includes bacterial vegetative forms, Gram-negative bacteria, Gram-positive bacteria (including MRSA, ORSA, VRSA, VRE), fungi, viruses, and protozoa. It is also thought that octenidine may be effective against the SARS-CoV-2 virus, which may be of practical importance due to its strong replication in the throat in the first days of the disease [11].

A review of recent scientific literature reveals an intensive development of research in biological and clinical studies on the action and innovative uses of cationic surfactants, including octenidine [12,13], and in chemical studies. So far, no confirmed cases of acquired resistance to this antiseptic have been reported [14].

Considering also the large production of cationic surfactants, there is an increasing need for the development of analytical methods to monitor them and control their content in different environmental objects or medicinal products for different purposes. Many of them are colourimetry-based methods [15,16] in which CS reacts with anionic dyes, resulting in complexes that are extracted into the organic solvent phase. However, these methods are not accurate in many cases because the surfactants often react with the sample matrix—as the affinity of CS for anionic surfactants, often present in samples, is commonly greater than their affinity for the dyes. Other methods for the quantification of cationic surfactants are also found in the literature, i.e., capillary electrophoresis [17], chromatography [18,19,20], and electrochemical methods [21]. These methods have many advantages, i.e., they are fast and simple, but their accuracy (apart from chromatographic techniques) is highly dependent on the sample matrix. They are not highly selective for individual cationic surfactants. In addition, they require expensive apparatus and time-consuming sample preparation.

One of the first and still currently used methods is the determination of CS by titration methods with both visual and potentiometric end-point detection [22], especially as pharmacopoeia methods are mainly based on this technique. Detailed information on the kinds of CS titration is included in the above review article; these are direct, reverse and two-phase titration methods. The last one is still a widely, commonly applied method which uses a suitable colour indicator, e.g., methylene blue, which forms an ion pair with the surfactant as a result of extraction with an organic solvent. As the titration is carried out, the titrating surfactant (the corresponding anion) forms an ion pair with the CS and the dye is displaced into the aqueous layer due to its insolubility in the organic solvent. Although two-phase titration has been used for many years, the EP (endpoint) of titration determination is subjective, especially in unclear samples, and requires large amounts of organic solvent, e.g., chloroform, which is associated with a negative environmental effect and high hazardous waste disposal costs.

Therefore, new analytical methods are constantly being developed as an improved alternative to the determination of cationic tensides. One such method is direct (potentiometric titration) and indirect potentiometry using ion-selective electrodes.

Potentiometry is a method of electrochemical analysis that measures the potential of a system consisting of two electrodes, a reference electrode, and an indicating electrode. The reference electrode has a constant potential that is reversible under given conditions, while the indicating electrode is an electrode with a variable potential depending on the concentration of the analyte. Potentiometric sensors (ion-selective electrodes (ISE)) measure the potential difference between two electrodes under no-current conditions.

The scheme of such a cell can be represented as follows:

Ag, AgCl inner solution membrane sample solution electrolyte key solution reference electrode.

The most important element of the electrode is the ion-exchange membrane (the receptor part of the sensor), in which ions interact with the active substance, known as the ionophore. At the interface between the membrane and the sample solution, a potential difference occurs, which depends on the activity of the ion present in both the membrane and the solution and is transferred from one phase to the other. The membrane potential (E_M_) is obtained as a result of the ion exchange reaction between the membrane and the sample solution (Equation (1)).

E_M_ = E_const_ + E_PB_ (1)

where E_PB_ is the phase boundary potential between the membrane and the solution, E_const_ is the sum of the phase boundary potentials and the diffusion potential.

In a state of equilibrium, the electrochemical potentials of molecules in the aqueous phase (aq) and in the organic phase (org) are equal. The equation for the phase boundary potential between the membrane and the solution is as follows:

(2) $$E_{PB}=\Delta \varphi =-\frac{\mu_{org}-\mu_{aq}}{zF}-\frac{RT}{zF}\ln a_{i(org)}+\frac{RT}{zF}\ln a_{i(aq)}$$

where Δϕ—electrical potential difference, μ—chemical potential, z—ion charge, F—Faraday constant, R—gas constant, T—temperature, a_i_—ion activity.

As a result of mathematical transformations and replacing the term a_i(org)_ and other values independent of the sample composition with a single constant term E_0_, the equation reduces to a relationship between the membrane potential and the logarithm of the activity of the ions being measured, known as the Nernst Equation (3):

(3) $$E_{M}=E_{0}+\frac{RT}{zF}\ln a_{i(aq)}$$

This equation applies to ideal conditions, i.e., a solution containing one type of ion. In practice, the activity of all types of ions present in the sample solution must be taken into account using the semi-empirical Nikolsky-Eisenman equation:

(4) $$E=E_{0}+S\log\left[a_{A}+\sum K_{A,B}^{pot}\right]{a_{B}}^{\frac{z_{A}}{z_{B}}}$$

where S—slope of the characteristic curve, a_A_, a_B_—activity of the main and interfering ions, z_A_, z_B_—charge of the main and interfering ions, $K_{A,B}^{pot}$—potentiometric selectivity coefficient [23].

Ion selective electrodes offer the possibility of simple, simultaneously accurate and precise measurement of the ionic concentration of an active compound. They are sensitive, selective and have a short response time. The entire analysis does not require toxic solvents, expensive apparatus, or a very long time. In addition to the above advantages, they are compatible with the analytical sample, do not require sample modification, and, unlike other analytical methods, the sensors are placed in the examined medium, which in a way imposes a small, concise design on them. The review article, which summarises papers that were published in the last two decades [24], is evidence of how the development and use of ion-selective electrodes for the determination of cationic surfactant groups are very common. It also demonstrates the continuing trend of this research.

A number of interesting electrodes with liquid and solid contact have recently been developed for the determination of cationic compounds, i.e., benzethonium chloride (Hyamine) [25], hexadecyltrimethylammonium bromide (CTAB) [26], cetylpyridinium chloride (CPC) [27], 1,3-didecyl-2-methylimidazolium chloride (DMIC) [28], dodecyltrimethylammonium (DTA) [29], or Septonex [30]. These electrodes are usually characterised by a theoretical sensitivity of about 58–60 mV/decade, or a super Nernstian response [26] in the linear range of 10^−2^, 10^−3^–10^−6^, and 10^−7^ M, while the response time is in the range between 30 and 60 s. The lifetime varies depending on the sensor design and ranges from one month to one year, or they can be disposable sensors. Potentiometric analysis of these compounds in commercial preparations can obtain analytes in the range of about 95–103%. From the literature review, UV-Vis spectrophotometric and Reverse-Phase High Performance Liquid Chromatography methods (RP-HPLC) [31] can be mentioned for the determination of octenidine among other few instrumental methods. So far, no ion-selective electrode has been made to determine octenidine dihydrochloride.

The present study, therefore, attempted to develop sensors that would selectively interact with the octenidine ion. The aim of the research was to achieve an optimised polymeric membrane composition and to study the analytical parameters of the electrodes, such as selectivity, measuring range, slope of characteristics, reversibility, potential drift, response time, pH range, and lifetime. Different designs were used in the study: liquid contact electrode (ISE), polymer electrode (PVC), and glassy carbon electrode (GCE). The electrode based on the cation exchanger, tetrakis(p-chlorophenyl)borate, had the best performance and was used to determine the active compound in synthetic and real samples.

## 2. Materials and Methods

### 2.1. Reagents, Pharmaceutical Formulations

The following reagents were used for membrane preparation: 2-nitrophenyloctyl ether (NPOE) (Fluka, St. Gallen, Switzerland), poly-vinyl chloride (PVC), (Tarwinyl, Tarnów, Poland), potassium tetrakis(p-chlorophenyl)borate (KTpCPB), sodium tetrakis [3,5-bis(trifluoromethyl)phenyl]borate (NaFPB), and heptakis (2,3,6-tri-O-benzoyl)-β-cyclodextrin (HSBβCD) (Sigma-Aldrich, St. Louis, MO, USA). The interferents, such as potassium chloride (KCl), were from Chempur, Poland, sodium chloride (NaCl) and sodium tartrate were from Fluka, St. Gallen, Switzerland, while citric acid and other interfering substances were obtained from Chempur, Poland. The following other reagents were applied: octenidine dihydrochloride (OCT) (Angene), tetrahydrofuran (THF) (POCh, Gliwice, Poland), acetic acid, sodium acetate, hydrochloric acid, and sodium hydroxide from (POCh Gliwice, Poland). All chemicals were of analytical—reagent grade. All aqueous solutions were prepared with deionized water of conductivity 0.07 μS/cm (Elix Advantage System Mili-Q plus Milipore, Spittal an der Drau, Austria). For the study, the following pharmaceutical preparations were used: MaxiSeptic (1 mg/mL + 20 mg/mL octenidine dihydrochloride + phenoxy ethanol) skin spry, solution (ICN Polfa SA Rzeszów, Rzeszów, Poland), and Octeangin (2.6 mg) lozenges (Klosterfrau Berlin GmbH, Berlin, Germany).

Samples to determine octenidine were prepared as follows: a stock solution of OCT at a concentration of 10^−2^ M was prepared by dissolving an appropriate weight of octenidine dihydrochloride in deionised water using an ultrasonic bath for 20–25 min. The sample solution (pure) of 2 × 10^−5^ M was then prepared by diluting the stock solution in acetate buffer, pH 5.8, prepared according to the procedure [32].

Octeangin samples were prepared as follows: 10 tablets of OCT 2.6 mg were weighed and then ground in a mortar. The amounts of the powder corresponding to the active substance of two tablets were weighed and dissolved in 100 mL deionised water, obtaining a solution with a concentration of 8 × 10^−5^ M. The sample solution (2 × 10^−5^ M) was prepared by diluting this solution and filling to the mark with buffer acetate of pH 5.8.To prepare the MaxiSeptic sample, 5 mL of the preparation was diluted into a 100 mL volumetric flask and filled to the mark with acetate buffer of pH 5.8.

### 2.2. Potentiometric Measurements, Apparatus, Analysis Procedure

Electromotive force measurements of the chlorhexidine electrode–reference electrode (Orion 90-02) system were carried out at 23 ± 1 °C using an Electrochemistry EMF Interface (Lawson Labs Inc., Malvern, PA, USA) connected to an IBM PC. pH values were determined with a Thermo Orion 81–72 (NTL, Warsaw, Poland) glass electrode and a CX–721 Elmetron multi-functional metre (±0.1 mV, Zabrze, Poland). During potentiometric experiments, the solutions were continuously stirred with a magnetic stirrer (IKA, Warsaw, Poland). Prior to the initial measurement, PVCE and GCE electrodes were conditioned for 48 h in a 10^−2^ M OCT solution. Before each subsequent measurement, electrodes were immersed for approximately 10 min in a 5 × 10^−5^ M OCT solution.

For electrode calibration, OCT standard solutions were prepared in the concentration range of 10^−6^–10^−3^ M by diluting a 10^−2^ M basic solution prepared from a suitably weighted sample. The electrodes were calibrated as follows: they were immersed in 50 mL of a 10^−3^ M solution and the potential was recorded after 5 min. The electrodes were then rinsed, dried, and immersed in a solution with a concentration of 10^−4^ M. The same procedure was followed for the next solutions. The EMF system allows for multiplexing of 16 electrodes. Thus, changes in the potential were recorded simultaneously as a function of the logarithm of the octenidine dichloride concentration for several sensors.

The analysis procedure for evaluating selectivity was similar. First, calibration curves for the electrodes in the main ion (OCT) solutions were determined according to the procedure described above. Then, calibration curves in the solutions of interfering ions and substances were determined. For this purpose, specific volumes of concentrated interferent solution were added to 50 mL of deionized water, and then the potential was recorded 5 min after each portion of the solution was added. As a result of titration, solutions with concentrations of 10^−6^, 10^−5^, 10^−4^, and 10^−3^ M were obtained. After calibration of the main ion solution and the specific interfering ion solution, the electrodes were rinsed with deionized water and then soaked for 5 min in deionized water to remove compounds from the membrane.

In order to examine the response time of the tested sensors, 50 mL of OCT solution with a concentration of 10^−4^ M was added to the beaker and then, after 5 min of potential measurement, 4 mL of OCT solution with a concentration of 10^−2^ M was added. The potential of the tested electrodes was measured for the next 5 min.

Potential reversibility (dynamic response) was determined by measuring the electrode potential over time. The electrodes were immersed in solutions with concentrations of 10^−3^, 10^−4^, 10^−5^, 10^−6^ M, and then in solutions with concentrations of 10^−5^, 10^−4^, 10^−3^ M. The time for a single potential measurement in a specific solution was about 7 min. After recording each potential, the sensors were rinsed and dried.

The analysis procedure used to test pH dependence was as follows: a sodium hydroxide solution, 10^−1^ M and then 10^−2^ M, was added in small drops to 50 mL of the tested octenidine solution of a concentration of 10^−4^ M until a pH of approx. 10 was obtained. A similar measurement was performed for a higher hydrogen ion concentration, using a 10^−2^ and 0.5 M hydrochloric acid solution as an additive. pH changes were measured using a pH metre with a glass electrode, while changes in potential were simultaneously recorded using an EMF metre.

### 2.3. Electrode Construction

**Figure 2:**
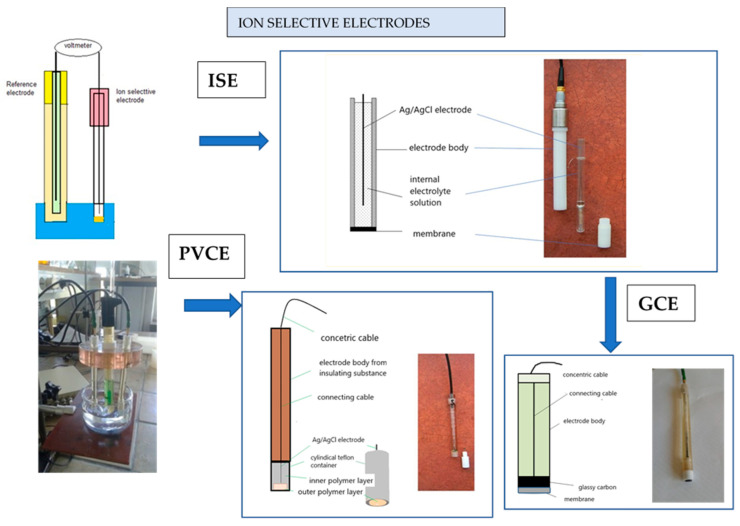
Schematic and image of ion-selective electrodes used in the study: ISE, PVCE, and GCE. From the left, a diagram and photo of the measuring cell. (Department of Analytical Chemistry UMCS University).

The electrode construction design is very simple, being previously reported in other publications [33,34,35], and is presented in Figure 2. The liquid contact electrode (ISE) contained an internal solution 1 × 10^−2^ M OCT + 1 × 10^−3^ NaCl M. The external solution was 1 × 10^−3^ M OCT. Electrodes of this type are described widely in the literature [33].

In polymer electrodes, the inner phase consists of a homogeneous mixture of plasticizer (modifier) and PVC (matrix), within which a reference Ag/AgCl electrode is placed. The potential of this reference electrode remains stable and is influenced by chloride ions generated during PVC degradation, as well as by the dissociation and partial dissolution of AgCl in the plasticizer. The process of plasticization of PVC takes place at temperatures above its glass transition point. The outer layer, which is in direct contact with the test solution, contains the active ingredient in addition to the components of the inner layer. This layer is prepared using the solvent evaporation method, where membrane components are dissolved in a low boiling solvent, most often tetrahydrofuran, and applied onto the pre-gelated inner layer. In this case, plasticization occurs below the glass transition temperature of PVC. The same method is also employed in the fabrication of conventional and glassy carbon electrodes.

The preparation of a polymer electrode with an Ag/AgCl inner reference electrode requires more time, but its support and use are much simpler, such as in the case of a glassy carbon electrode. The preparation of the PVC electrode consisted of several stages: (1) obtaining an Ag/AgCl inner reference electrode by electrolysis in a concentrated hydrochloric acid solution; (2) weighing the inner layer components (PVC+ NPOE); (3) mixing and deaerating the obtained mixture by means of a vacuum oil pump; (4) filling the Teflon sensor with the mixture to cover the inner electrode; and (5) gelating the layer at a temperature of 353 K for 30 min.

The finished bodies filled with glassy carbon were prepared by polishing the surface with a suspension of aluminium oxide hydrated with deionized water for about 5 min and rinsing them with water in an ultrasonic bath for about 15 min. The electrode surfaces were then defatted and dried using tetrahydrofuran (THF) for 5 min. A membrane cocktail was then dripped onto the prepared glassy carbon surfaces.

### 2.4. Membrane Preparation

Three membrane compositions were prepared for the constructed electrodes. These compositions are presented in Table 1. The components of the PVC-based membrane for all the electrodes contain polyvinyl chloride (30% *w*/*w*), plasticizer (65% *w*/*w*), ionophore (3% *w*/*w*), and lipophilic salt (2–5% *w*/*w*). These components were dissolved in 2 mL of tetrahydrofuran.

For the ISEs, the mixture solution was poured into a glass ring of 30 mm in diameter placed on a glass plate and left for the solvent to evaporate at room temperature for about 12 h. Next, small membranes of approx. 5 mm in diameter were cut out and installed in a Teflon sensor housing, made by Philips IS-561 (Merck Darmstadt, Germany). In the case of the PVCE and GCE electrodes, the membrane cocktail was applied to the surface of the inner electrode (4 × 50 μL, PVCEs, and 3 × 10 μL, GCEs). After the first calibration, the solid contact electrodes were stored in air between the measurements.

## 3. Results and Discussion

### 3.1. Characterisation of Octenidine Ion Selective Electrodes by Potentiometric Response

A crucial step in electrode preparation is the optimisation of the ion-selective membrane, including both the choice and proportion of its components. Various strategies are possible, such as using commercially available ionophores, synthesing new compounds, applying different plasticizers or their mixtures, and selecting an appropriate supporting matrix. Typically, a polymer membrane consists of 60–70% (*w*/*w*) plasticizer, about 30% (*w*/*w*) PVC, and 1–5% (*w*/*w*) ionophore, sometimes with a small amount of lipophilic salt acting as an ion exchanger. The polymer matrix, most commonly PVC, provides mechanical stability while remaining chemically inert. Owing to the high diffusion coefficient of low-molecular-weight ionophores (≈10^−^^8^ cm^2^/s), such membranes can be considered as liquids. Plasticized PVC is widely used because it combines good mechanical strength resulting from its structure, with sufficient mobility of the active components (ionophore, exchanger, ion-pair complex). Although ion transport in the membrane is about one thousand slower than in water, this system is advantageous due to its simplicity, availability, and low cost [24,33]. The plasticizer, which is the main membrane component, reduces viscosity, facilitates ion transport, and influences both electrode performance and lifetime. The most commonly used plasticizers are o-nitrophenyl octyl ether (o-NPOE), bis(2-ethylheksyl)sebacate (DOS), dibutyl phthalate (DBP), and bis(2-ethyl-hexyl) adipate (DOA).

For cation-selective electrodes responsive to cationic surfactants, ion exchangers are most frequently applied. As an alternative, widely available macrocyclic compounds, including crown ethers and cyclodextrins, can also be employed [36]. The latest studies on chlorhexidine electode of the author [37] focused on the qualitative optimisation of the polymeric membrane. Based on these results and literature data [38], two ion exchangers, KTpCPB, NaFPB, a neutral ionophore from the cyclodextrin group HSBβCD, and o-NPOE as solvent were chosen for the octenidine electrode study. The obtained membrane compositions are shown in Table 1. In the first research step, three different membranes were used in the classical construction (ISE 1, ISE 2, ISE 3). The selected membrane compositions were then used to make polymer electrodes with an Ag/AgCl conducting electrode (PVC 1, PVC 2, PVC 3) and glassy carbon electrodes that contained two different membranes (composition 2 and 3). Several electrode calibration measurements were performed and the parameters obtained from the response curves are summarised in Table 2.

**Figure 3:**
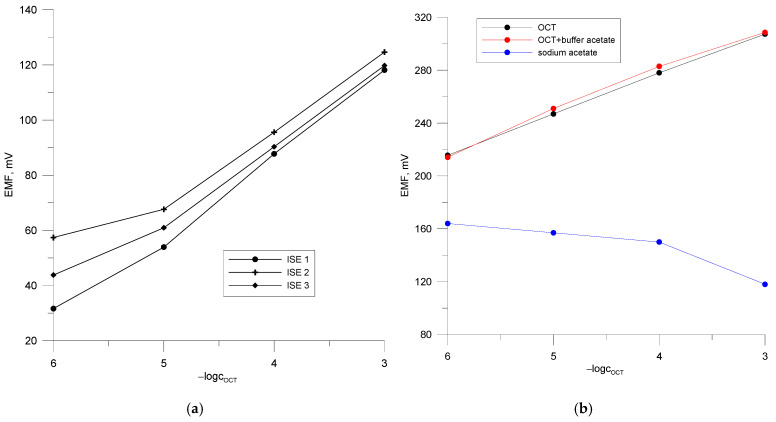
Calibration curves of the ISE electrodes in the octenidine dihydropchloride solutions. (**a**) Calibration curves of electrode PVCE 2 in the water and buffered solution of octenidine dihydrochloride and in the CH_3_COONa solutions; (**b**).

The classical electrodes (ISE 1, ISE 2, ISE 3, Figure 3a) show a very similar characteristic slope of 28–30 mV/decade in the linear range of 10^−3^–10^−5^ M with a high linear correlation coefficient of 0.9999. The detection limit is about 10^−6^ M. The experimental data were recorded over a period of 1 week. The achieved electrode parameters prove that the qualitative and quantitative composition of the ion-selective membrane was correctly selected and that it can be used in further steps of the research work to construct PVCE and GCE electrodes. Although classical ion-selective electrodes with an internal solution were already developed in the last century, they also have relatively many disadvantages in use, i.e., they have to be stored in vertical position and require an external solution for storing the electrodes between the measurements. The lifetime of these electrodes is also much shorter, but they are still used today, especially in studies on new undetermined compounds, in membrane composition studies, or for the determination of compounds by potentiometric titration [38,39,40].

The parameters of the polymer electrodes (Table 2) were determined over a one-month time span. The slope of the characteristics for all the three electrodes is within the Nernstian range of 27–32 mV/decade, with a much wider concentration range of 10^−6^–10^−3^ M for electrodes 2 and 3. The detection limit is therefore also lower, about 5 × 10^−7^ M. The preparation time for these electrodes is slightly longer than for the ISE electrodes (3 to 4 days) and especially the first inner layer requires the ingredients to be weighed, while the mixture to be deaerated, gelled and cooled. But the use is simpler and more convenient, the electrodes are stored dry, in any position, and the lifetime of these electrodes is longer. The properties of electrode PVCE 2 are comparable to those of electrode PVCE 3, but for economic reasons (the membrane with the exchanger alone is cheaper than the membrane with cyclodextrin), it was optionally chosen for further selected studies. The GCE sensors were prepared in 1 day and in this case the labour input is the lowest. As can be seen from the experimental data (Table 2), both sensors show the same good, similar analytical performance, a Nernst slope of 29.8 mV/decade in the concentration range −logc = 3–5, and a detection limit of 2 × 10^−6^ M.

To obtain accurate results from ion concentration determinations using an ion-selective electrode, the composition of the sample matrix as well as the calibration solutions should be the same. For this purpose, a stock electrolyte was selected that provided a high constant ionic strength of the solutions. As an example, the calibration of PVCE 2 in sodium acetate solutions, in pure octenidine, and octenidine solutions in acetate buffer, pH 5.8, is shown in Figure 3b. The experimental data show that the electrode is selective for octenidine relative to acetate. In the pure drug solution, it has the following parameters: S = 30.62 mV/decade R^2^ = 0.9998, E^0^ = 399.74 mV. The linear range is −logc = 3–6. In a mixed solution of octenidine and acetate buffer, the sensor shows similar characteristics as in the pure solution: a slope of 28.8 mV/decade over a linear range of 10^−3^–10^−5^ M, with R^2^ = 0.9961 and E^0^ = 396.03 mV. It can be assumed that the sodium acetate buffer solution at pH 5.8 is a suitable standard electrolyte solution for the determination of octenidine concentration. It can therefore be concluded that the activity coefficients of the octenidine ion in all sample and standard solutions are the same.

### 3.2. Selectivity

**Figure 4:**
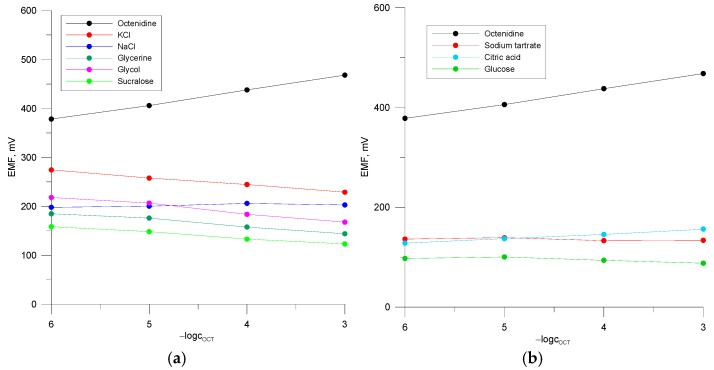
(**a**,**b**) Calibration curves of the three-month electrode PVCE 2 in the main and interfering ion solutions.

Selectivity is one of the most important parameters of ion-selective electrodes, which influences significantly the results of ion determination in a sample. It was therefore decided to determine the selectivity of all the three types of electrode, as regards their construction: polymeric (PVCE 2), glassy carbon (GCE 2), and classical electrode. Selectivity coefficients were determined in relation to inorganic cations and most excipients occurring in pharmaceutical preparations with octenidine. The values of the selectivity coefficients were determined using the separate solution method (SSM). For this purpose, calibration curves were recorded in the concentration range of the main and interfering solutions 10^−6^–10^−3^ M (Figure 4) and selectivity coefficients were determined by extrapolating the response functions to c_I_ = c_J_ = 1 M according Bakker’s method (Equation (5)) [41]. In the solutions of most interfering compounds, apart from NaCl (Figure 4a,b), no potential changes were observed, or the changes were very small in relation to the anionic function. Therefore, for some interferents $E_{J}^{0}$ values were determined by extrapolation of a portion of the characteristic (from the concentration of −logc 6−5, for potassium glycerine, glycol, and sucralose). It can be seen from Table 3 that the selectivity coefficients are, for most ions, generally very similar and favourable for all the electrodes. Importantly, smaller values for inorganic ions and other excipients are achieved for electrode PVCE 2. The sequence of $K_{I,J}^{pot}$ for ionic substances for electrodes PVCE 2 and GCE 2 is as follows: K^+^ > Na^+^ > sodium tartrate >citric acid. In the group of excipients, the values of the selectivity coefficients decrease in the following order: glycol > glucose > sucralose > glycerine and glycol > glycerine > sucralose > glucose for electrodes PVCE 2 and GCE 2, respectively.

(5) $$\mathrm{Log}K_{I,J}^{\mathrm{pot}}=\frac{{E_{J\;}^{0}\;-\;E}_{I}^{0}}{\mathrm{RT}}\mathrm{zF}\;$$

### 3.3. Response Time, Reversibility and Potential Drift

**Figure 5:**
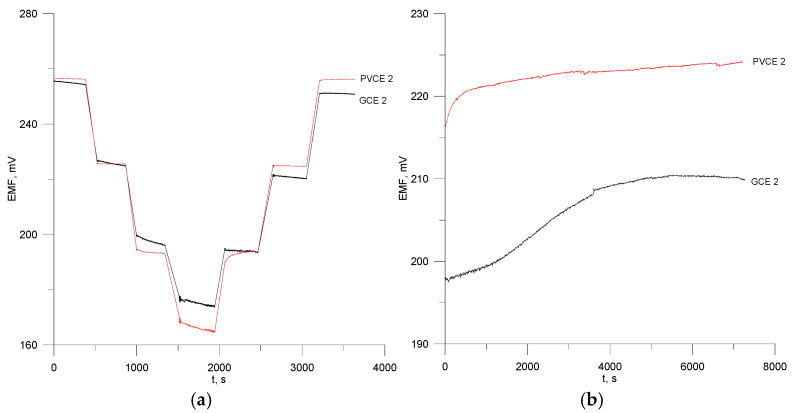
Dynamic response of the electrodes PVCE 2 and GCE 2 in the water solution of octenidine dihydrochloride (**a**) Potential stability of electrodes no. PVCE 2 and GCE 2 (**b**).

The time-dependent parameters, including response time, potential reversibility and potential stability, were determined. In order to evaluate the reversibility of the potential, measurements were carried out in solutions with different octenidine dihydrochloride concentrations. The calibration was carried out in the concentration range of 10^−^^6^–10^−^^3^ M, first decreasing and then increasing the concentrations, as shown in Figure 5a. The figure shows that the polymer electrode has slightly better potential reversibility in the solutions with higher concentrations, i.e., 10^−3^, 10^−^^4^ and 10^−5^ M. The mean values of the potential of electrode PVCE 2 were 256.2 ± 0.3 mV, 225.2 ± 0.7 mV and 193.0 ± 0.7 mV in the OCT solutions 10^−3^, 10^−4^, and 10^−5^ M, respectively. In the case of the electrode based on glassy carbon, the average potential, with standard deviation, in these solutions was 252.7 ± 3.5, 223.2 ± 4.5, and 196.0 ± 3.8, respectively.

A measurement was made to determine the short-term stability of the octenidine electrode potential when soaking the electrodes in a 5 × 0^−4^ M OCT solution for 2 h. The results obtained are presented in Figure 5b. The potential drift determined from ΔE/Δt was 60  µV/min and 100 µV/min for PVCE 2 and GCE 2, respectively.

**Figure 6:**
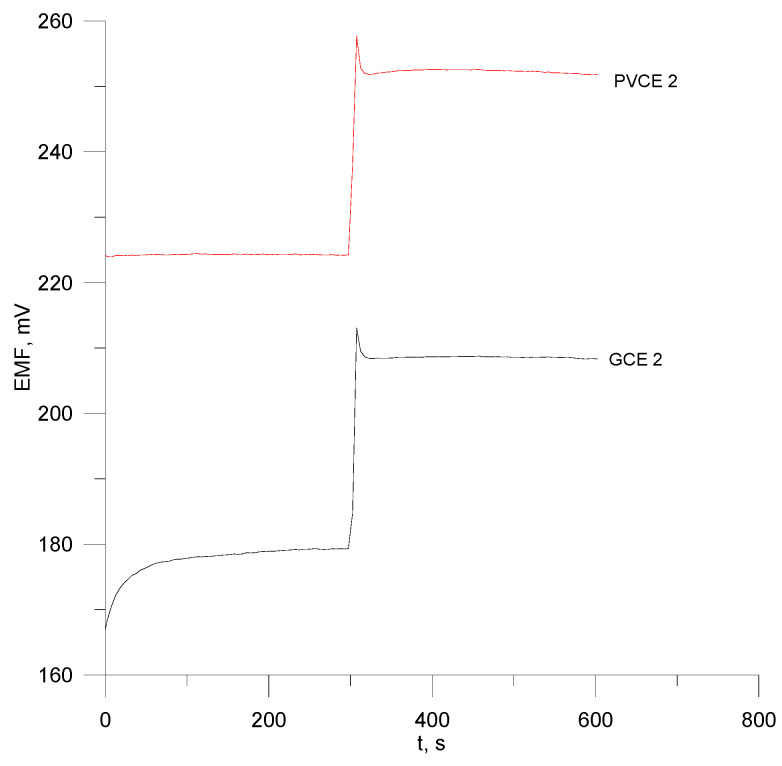
Response time of electrodes no. PVCE 2 and GCE 2.

The response time for the octenidine electrodes (Figure 6) was determined by the method of injecting a concentrated standard solution of a concentration of 10^−2^ M into the intensively stirred octenidine sample solution of a concentration of 10^−4^ M. The results presented in the graphs can give information about the response time of the two electrodes when concentrating the sample, which is approximately 5–8 s.

### 3.4. pH Dependence

**Figure 7:**
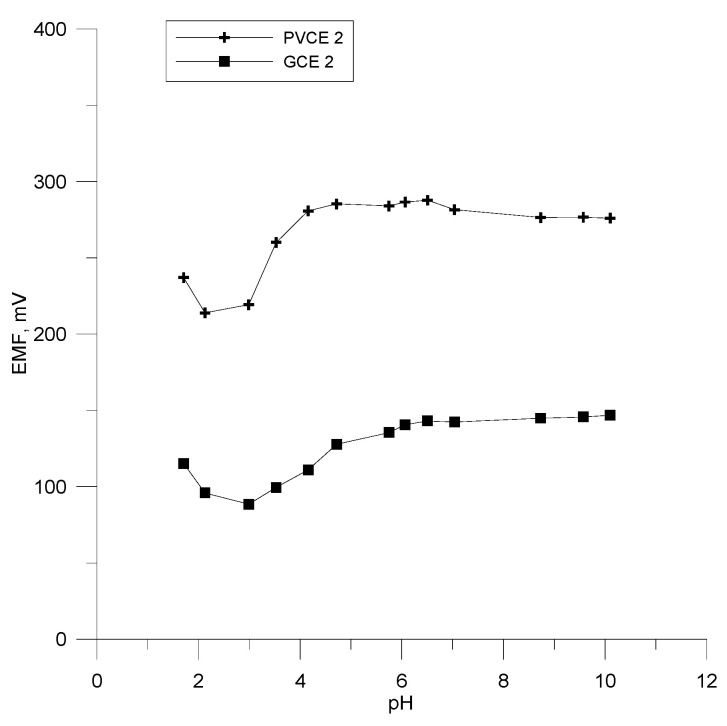
The effect of pH on electrodes PVCE 2 and GCE 2.

The pH dependence of the examined polymer electrodes was determined using a solution of 10^−4^ M OCT and moreover HCl and NaOH solutions as additives. The dependence of the potential of the tested electrodes was measured in the pH range of 1.5–10 and it is shown in Figure 7. The results show a stable potential response for the pH range of 4.0–9.5. In acidic medium (pH < 4), the interference effect from H^+^ ions is visible, while the increase that takes place at pH values higher than 9.5 is most probably due to the formation of free octenidine base in the test solution.

### 3.5. Potentiometric Determination of Octenidine

The analytical usefulness of the octenidine electrode based on KTpClPB was successfully confirmed for the determination of octenidine dihydrochloride salt in pure samples (Angene) and the pharmaceutical preparations MaxiSeptic and Oceangin (Table 4). The determination was made by the calibration curve and standard addition methods. The results of OCT determination in synthetic solutions aimed at verifying the correct functioning of the sensors. For PVCE 2, 101–102% of the compound was obtained. The results of API determination in the pharmaceutical preparation “MaxiSeptic” (ICN Polfa) using the potentiometric method provided the least positive results in terms of accuracy and precision (SD > 3 mg/L). The recovery from 3 replicate measurements for the determination of API in Octeangin, found to be 95–97%, and the precision (SD 2.3–2.8 mg/L) for PVCE 2 were quite good and acceptable.

The results obtained indicate a recovery in agreement with the requirements of the Pharmacopoeia, according to which uncoated tablets containing less than 100 mg have an acceptable deviation of ±10%. The electrode shows typical accuracy and precision for potentiometric methods and may therefore find practical use for the determination of octenidine in aqueous samples. The results of octenidine dihydrochloride determination obtained using the proposed method were compared with the results obtained by UV spectrophotometry and RP-HPLC method. The experimental data are presented in Table 4. For these methods, the precision and accuracy are slightly better than for the potentiometric electrodes.

## 4. Conclusions

In this work, a sensor with solid contact for octenidine determination was proposed. The suitability of the proposed polymer membrane components was evaluated on the basis of the constructed classical ion-selective electrodes. All the three membranes were found to have similar calibration curves. Next, polymer sensors were constructed which showed slightly better performance and a wider linearity range of 10^−^^6^–10^−^^3^ M, with a good theoretical characteristic slope. The sensors were tested over a period of 6 months and still function correctly, keeping a repeatable response with a deviation of S ± 16% mV/decade.

The best electrode that can find practical application is PVCE 2, characterised by a wide linear range, a low limit of detection, and a near-Nernstian slope of characteristics with small standard deviation. A stable, reversible potential and a short response time was achieved for this sensor. The obtained favourable selectivity coefficients of the electrode determined in relation to excipients allow direct determination of OCT, e.g., in a sample of lozenges, with the accuracy and precision typical of potentiometric methods. The proposed electrode is very convenient and cheap to use, can be stored in the air, and does not require vertical position storage. It is characterised by good mechanical resistance and is a self-acting sensor. Due its design, this electrode has a relatively long lifetime. The absence of an internal reference solution reduces elution of the membrane components. Moreover, the electrode can be quickly and easily prepared from commonly available and inexpensive materials. As an alternative to this electrode, a well-acting glassy carbon-based electrode may also be proposed, but with slightly more expensive materials. This electrode (GCE2 and GCE 3) is the aim of further research of the author. Research will be planned in the following areas: modification of the membrane phase, introduction of a mediation layer of suitable materials, and verification of the electrical properties of the (achieved) membranes. The planned research may lead to electrodes with even better properties, i.e., potential, reversibility, and stability. It is also worth mentioning that drug-sensitive electrodes are not commercially available and can only be obtained in a laboratory. The approximate price of commercially available ion-selective electrodes is about tenfold the price of laboratory-made electrodes from purchased materials.

## Figures and Tables

**Table 1:** Composition of the membrane phase of the prepared ion-selective electrodes.

Membrane Composition	PVC, mg	NPOE, mg	NaFPB, mg	KTpClPB, mg	HSBβCD, mg
1	130	60	10	-	-
2	130	60	-	10	-
3	130	60	-	4	6

**Table 2:** Analytical parameters of the designed ion-selective electrodes: classical, polymeric and glassy carbon, together with standard deviation values.

Electrode Symbol	Slope S ± SD, mV/Decade	*n*	Linear Range −logc, M	Standard Potential E^0^ ± SD, mV	Correlation Coefficient, R^2^ ± SD	Limit of Detection, M	Time of Measurements *
ISE 1-NaFPB	30.15 ± 1.61	3	5–3	216.42 ± 2.09	0.9980 ± 0.0016	1.25 × 10^−6^	
ISE 2-KTpClPB	28.38 ± 0.34	3	5–3	210.00 ± 1.10	0.9999 ± 0.0000	3.55 × 10^−6^	1 week
ISE 3-KTpClPB + HSBβCD	28.89 ± 0.88	3	5–3	209.65 ± 0.84	0.9999 ± 0.0002	1.41 × 10^−6^	
PVCE 1-NaFBP	27.54 ± 2.69	4	5–3	314.36 ± 29.15	0.9977 ± 0.0017	7.94 × 10^−6^	
PVCE 2-KTpClPB	31.41 ± 1.14	4	6–3	381.12 ± 45.17	0.9990 ± 0.0009	5.01 × 10^−7^	1 month
PVCE 3-KTpClPB +HSBβCD	32.38 ± 2.22	4	6–3	206.19 ± 48.78	0.9960 ± 0.0041	7.07 × 10^−7^	
GCE 2-KTpClPB	29.83 ± 0.56	3	5–3	318.04 ± 13.87	0.9985 ± 0.0017	2.24 × 10^−6^	
GCE 3-KTpClPB + HSBβCD	29.86 ± 0.69	3	5–3	216.87 ± 19.84	0.9987 ± 0.0016	2.82 × 10^−6^	3 days

* Time of measurement of the analytical parameters from the calibration curves.

**Table 3:** Potentiometric selectivity coefficients of electrodes ISE 3, PVCE 2, and GCE 2 determined with the SSM method.

	$\mathrm{Electrode}\;\mathrm{Selectivity}\;\mathrm{Coefficients}\;K_{I,J}^{pot}$							
	K^+^	Na^+^	Glucose	Glycol	Glycerine	Sucralose	Sodium Tartrate	Citric Acid
ISE 3	8.4 × 10^−2^	7.6 × 10^−2^	6.6 × 10^−7^	3.2 × 10^−4^	5.6 × 10^−7^	1.2 × 10^−4^	8.9 × 10^−4^	1.0 × 10^−4^
PVCE 2	3.2 × 10^−3^	1.3 × 10^−3^	3.8 × 10^−7^	5.3 × 10^−7^	3.3 × 10^−7^	3.4 × 10^−7^	4.2 × 10^−4^	2.2 × 10^−4^
GCE 2	5.1 × 10^−3^	9.5 × 10^−4^	4.5 × 10^−7^	6.2 × 10^−7^	6.0 × 10^−7^	5.1 × 10^−7^	5.7 × 10^−4^	1.0 × 10^−4^

**Table 4:** Results obtained in the analysis of octenidine samples by the proposed ion selective electrode PVCE 2. (n = 3) and comparison with other methods.

Method	Taken Octenidine Concentration, mg/L	Found Octenidine Concentration ± SD, mg/L	Recovery, %	Relative Error, %
Potentiometry Calibration curve	12.5 (pure)	12.6 ± 0.6	100.8	0.8
	12.5 (Octeangin)	12.1 ± 2.3	97.0	3.2
Potentiometry Standard addition	12.5 (pure)	12.7 ± 0.6	102.2	1.6
	12.5 (Octeangin)	11.9 ± 2.8	95.8	4.8
	31.0 (MaxiSeptic)	32.0 ± 3.1	103.3	3.1
UV Spectrophotometry [30]	100 mg Octenisept	99.8 mg	99.8 ± 0.05%	
RP-HPLC [30]	100 mg Octenisept	99.6 mg	99.6 ± 0.05%	

## Data Availability

The original contributions presented in this study are included in the article. Further inquiries can be directed to the corresponding author.
